# Food Insecurity Is an Important Risk Factor for Alzheimer’s Disease: A Case-Control Study

**DOI:** 10.34172/jrhs.7824

**Published:** 2025-06-10

**Authors:** Mohammad mehdi Abbasi, Mahdyeh Karimi, Sajjad Khandae, Bahram Rashidkhani

**Affiliations:** ^1^Department and Faculty of Nutrition and Food Technology, National Nutrition and Food Technology Research Institute, Shahid Beheshti University of Medical Sciences, Tehran, Iran; ^2^Iran Dementia and Alzheimer’s Association, Tehran, Iran; ^3^Department of Community Nutrition, Faculty of Nutrition Sciences and Food Technology, National Nutrition and Food Technology Research Institute, Shahid Beheshti University of Medical Sciences, Tehran, Iran; ^4^Department of Health, Iran University of Medical Sciences, Tehran, Iran

**Keywords:** Food insecurity, Alzheimer’s disease, Dementia, Neurocognitive disorders, Diet

## Abstract

**Background:** There is a gap in the literature specifically addressing the relationship between food insecurity and the risk of Alzheimer’s disease (AD).

**Study Design:** A case-control study.

**Methods:** This study aimed to evaluate the association between food insecurity and the risk of AD in 301 participants, including 150 cases and 151 controls. Cases were recruited among people in the early stages of the disease who had been diagnosed with AD within the past six months. Controls were selected from health centers across Tehran. Food security status was assessed using the validated Persian translation of the 18-item United States Department of Agriculture’s household food security questionnaire.

**Results:** After adjusting for potential cofounders, food insecurity was associated with a higher risk of AD (odds ratio [OR]: 2.80; 95% confidence interval [CI]: 1.59–4.94). Among female participants, food insecurity increased the odds of AD (OR: 3.54; 95% CI: 1.72–7.30). For individuals under 65, food insecurity also increased the likelihood of AD nearly four-fold (OR: 3.94, 95% CI: 1.48–10.47), while for those over 65, the risk was only 2.89 times (OR: 2.89, 95% CI: 1.36–6.14).

**Conclusion:** Food insecurity might be associated with an increased risk of AD. Further research is required to explore the relationship between food insecurity and other types of neurological disorders or health conditions. More precisely, future studies should aim to investigate the association in a prospective design.

## Background

 Alzheimer’s disease (AD), the most common form of dementia, is a progressive neurological disorder marked by cognitive decline, memory loss, and behavioral changes.^1-3^ Its prevalence is rising globally with aging in populations.^4-6^ While the exact causes of AD remain unclear, research highlights environmental, genetic, and lifestyle factors, with nutrition emerging as a critical influence on brain health.^7-9^ Diet plays an important role in both the prevention and management of AD.^7,10^

 Food insecurity, a major global public health issue, particularly affects older adults.^11,12^ It refers to the inability to consistently access sufficient, nutritious food due to financial or logistical barriers.^12,13^ Beyond contributing to chronic conditions such as heart disease and diabetes, emerging research suggests food insecurity may also significantly impact the development and progression of cognitive disorders.^14-17^

 Recent studies have examined the impact of food insecurity on memory, dementia, and cognitive trajectories in older adults.^18-20^ However, there is a gap in the literature specifically addressing the relationship between food insecurity and the risk of AD. Research has shown that food insecurity is linked to faster declines in memory, executive function, and overall cognitive performance among older individuals.^18,21,22^ Additionally, a cohort study found that food insecurity is associated with a higher risk of dementia and poorer cognitive outcomes.^20^ Leung et al^19^ further reported that food insecurity is linked to a two-fold increase in the likelihood of developing probable dementia.

 Our paper explores the relationship between food insecurity and the risk of AD. More precisely, it investigates how limited access to food may contribute to the onset and progression of AD.

## Methods

###  Subjects

 This case-control study focused on AD patients admitted to the Iran Alzheimer and Dementia Association in Tehran, Iran, between September 2023 and June 2024. Participants were classified as AD patients if diagnosed by the center’s neurologist based on magnetic resonance imaging scans and the Persian version of the Modified Mini-Mental State Examination (3MS),^23^ with no history of other cognitive disorders. Eligible cases included all new AD diagnoses from the past six months, provided the caregiver was available for the interview. On the other hand, the exclusion criteria for AD patients were those whose disease had progressed beyond the fourth stage of the Reisberg Functional Assessment Staging Scale,^24^ caregivers without knowledge of the patient’s pre-diagnosis diet, and patients with specific dietary habits, such as being vegetarian.

 The control group was randomly selected from visitors at six health centers across the city of Tehran, ensuring that cases and controls were chosen from separate facilities. After an examination by the center’s physician, participants completed the 3MS questionnaire with a trained interviewer to rule out cognitive disorders. Controls were excluded if they had a history of any diagnosed cognitive disorder, a 3MS score below 78,^23^ or special dietary habits. Participants were matched by age and gender; a form of paired matching with 5-year age groups was used for age. The overall participation rate was 92%. Of the 305 eligible subjects, two cases and two controls were excluded due to missing data for weight, height, and body mass index (BMI). Ultimately, 301 subjects (150 cases and 151 controls) were included in the final analysis. The study protocol was approved by the Ethics Committee of the National Nutrition and Food Technology Research Institute at Shahid Beheshti University of Medical Sciences (approval No. IR.SBMU.NNFTRI.REC.1402.027), and written informed consent was obtained from all participants.

###  Food security 

 Food security status was assessed using the 18-item United States Department of Agriculture household food security questionnaire,^25^ which has been validated and is reliable in Iran.^26,27^ The scoring system for this questionnaire assigned a score of 1 for responses indicating food insecurity (e.g., “often”, “sometimes”, “almost every month”, “some months, and “yes”) and a score of 0 for responses representing food security (e.g., “not correct”, “refused or didn’t know”, “only once or twice a month”, and “no”). Based on the responses, food security was categorized into four levels, including food secure, food insecure without hunger, food insecure with moderate hunger, and food insecure with severe hunger (Table 1).^25^ For the purposes of this study, participants were grouped into food secure and food insecure categories. The food security score ranged from 0 to 18, with scores of 0–2 and 3–18 indicating food security and food insecurity, respectively.

**Table 1:** Household food security status based on positive responses to the USDA Food Security Questionnaire

**Food security status**	**Families with children under 18 (Total score: 18)**	**Families without children under 18 (Total score: 10)**
Secure	0-2	0-2
Insecure without hunger	3-7	3-5
Insecure with moderate hunger	8-12	6-8
Insecure with severe hunger	13-18	9-10

*Note*. USDA: United States Department of Agriculture. Guide to Measuring Household Food Security.^25^

###  Data collection 

 Trained interviewers conducted in-depth interviews with caregivers to collect data on various socio-economic and demographic factors, including the participants’ medical history (e.g., cognitive disorders and other health conditions), a family history of AD, and smoking habits. Physical activity levels were evaluated through the Persian version of the International Physical Activity Questionnaire, with results converted into metabolic equivalent scores (MET-min-week-1).^28^ Weight was measured using a digital scale (Seca, Germany) while participants were lightly dressed and barefoot, with measurements rounded to the nearest 100 g. Height was recorded using a wall-mounted stadiometer (Seca, Germany), accurate to 2 mm, with participants standing barefoot. BMI was calculated by dividing weight in kilograms by the square of height in meters. The economic score was determined based on family income, family size, furniture, leisure time activities, house dimensions, and homeownership status.^29^

###  Statistical analysis

 The statistical analyses were conducted using SPSS, version 27 (SPSS Inc., Chicago, IL, USA). Significance tests were conducted with a confidence interval (CI) of at least 95% (*P* ≤ 0.05). The normality of the variables was assessed using the Kolmogorov-Smirnov test, and the Chi-square test was applied to evaluate the relationships between categorical variables. In addition, the independent samples t-test was utilized to examine the association between continuous variables. Food security status (food secure and insecure) was analyzed as distribution-based indices, with the food secure status serving as the reference category. Unconditional logistic regression was used to estimate odds ratios (ORs) and their 95% CIs for food security status and the risk of AD in multivariate adjusted models. Subgroup analysis was performed to indicate the effect among female participants and different age groups (older than 65 and younger than 65 years old). These models were adjusted for significant variables identified between the two groups, including BMI (kg/m^2^), family size (1–2 persons or more than 2 persons), job status (housewife, retired, or employed), education (under diploma, diploma, or university education), number of children, and family history of AD.

## Results

Table 2 presents the demographic characteristics of the study groups. Participants in the control group had a notably greater number of children, weight, and BMI. In the case group, the number of participants with a family history of AD was significantly higher than that of the controls. Additionally, compared to the control group, more subjects experienced food insecurity among the cases. In the control group, a significantly higher proportion of participants held a diploma or university education compared to cases. Further, a significant difference was observed between cases and controls for family size and job status (Table 2).

**Table 2:** Baseline characteristics of 301 participants in a case-control study in Tehran

**Variables**	**Control (n=151)**		**Case (n=150)**		***P*** **value**^a^
**Quantitative variables**	**Mean**	**SE**	**Mean**	**SE**	
Age (year)	66.84	0.69	67.74	0.61	0.339
Weight (kg)	75.93	1.14	68.29	0.85	0.001
Height (cm)	164.11	0.83	161.31	0.73	0.011
BMI (kg/m^2^)	28.18	0.36	26.28	0.32	0.001
Physical activity (MET-minute/week)	3423	282	2815	247	0.107
Economic score	22.24	0.41	21.68	0.23	0.240
Number of children	2.81	0.15	3.85	0.16	0.001
**Qualitative variables**	**Number**	**%**	**Number**	**%**	***P*** **value**
Gender					0.941
Male	57	37.7	56	37.3	
Female	94	62.3	94	62.7	
Family history of Alzheimer’s					0.001
No	119	78.8	89	59.3	
Yes	32	21.2	61	40.7	
Marital status					0.158
Married	110	72.8	98	65.3	
Single/deceased spouse/divorced	41	27.2	52	34.7	
Food security					0.001
Secure	80	53.0	60	40.0	
Insecure					
Without hunger	66	43.7	63	42.0	
With moderate hunger	5	3.3	21	14.0	
With severe hunger	0	0	6	4.0	
Family size (persons)					0.023
1-2	76	50.3	94	63.3	
≥ 3	75	49.7	55	36.7	
Education					0.002
Under diploma	75	49.7	93	62.0	
Diploma	33	21.9	39	26.0	
University education	43	28.5	18	12.0	
Job status					0.001
Housewife	78	51.7	73	48.7	
Retired	35	23.2	64	42.7	
Employed	38	25.2	13	8.7	
High blood pressure	73	48.3	67	44.7	0.522
Diabetes	32	21.1	33	22.0	0.865

*Note*. SE: Standard Error; BMI: Body mass index. ^a^ Obtained from independent t-test, ^b^Obtained from chi-squared test

 The ORs and their 95% CI for food insecurity status and risk of AD are provided in Table 3. After accounting for potential confounders, a significant association was found between food security status and odds of AD with a *P* value of 0.002. Individuals experiencing food insecurity had an OR of AD that was 180% higher than those who were food secure (OR: 2.80, 95% CI: 1.59–4.94). Additionally, among female participants, food insecurity increased the odds of AD by 3.54 times (OR: 3.54, 95% CI: 1.72–7.30). Additionally, the results indicated that those under the age of 65 with food insecurity were nearly four times more likely to develop AD compared to those who were food secure (OR: 3.94, 95% CI: 1.48–10.47). Finally, among participants older than 65 years old, those with food insecurity had an OR of AD that was 2.89 times higher than those who were food secure.

**Table 3:** Crude and Adjusted ORs (95% CIs) for Alzheimer’s Disease by Food Security Status

	**Cases**	**Controls**	**Crude OR (95% CI)**	**Adjusted OR (95% CI)**^a^
Food security status				
Secure	60	80	1.00	1.00
Insecure	90	71	1.69 (1.07–2.67)	2.80 (1.59–4.94)
*P* trend			0.024	0.001
Women				
Secure	39	55	1.00	1.00
Insecure	55	39	1.99 (1.11–3.55)	3.54 (1.72–7.30)
*P* trend			0.020	0.001
Early onset dementia ( < 65 years)				
Secure	31	42	1.00	1.00
Insecure	29	33	1.19 (0.62–2.35)	3.94 (1.48–10.47)
*P* trend			0.616	0.006
Older than 65 years				
Secure	29	38	1.00	1.00
Insecure	61	38	2.10 (1.12–3.95)	2.89 (1.36–6.14)
*P* trend			0.021	0.006

*Note*. BMI: Body mass index; OR: Odds ratio; CI: Confidence interval. ^a^ Adjusted for BMI, family history of Alzheimer’s, family size, job status, education, and number of kids.

## Discussion

 This observational study explored the relationship between food security and the risk of AD. Our analysis showed that food insecurity is significantly associated with an increased risk of AD. After adjusting for confounders, the association between food insecurity and AD risk remained significant (*P* < 0.01). Among women, the risk was even greater, with food insecurity increasing the odds of AD. For individuals over 65, food insecurity could significantly increase the likelihood of AD, while for those under 65, the association was only significant in the adjusted model.

 The results demonstrated that individuals experiencing food insecurity had greater odds of developing AD compared to those with food security. In line with our findings, Qian et al^20^ indicated that older adults with low or very low food security had a 1.37–1.38 times greater likelihood of developing dementia compared to those who were food secure. Moreover, in this cohort study, food security was associated with reduced baseline memory levels at age 70 and accelerated memory decline over time. Low and very low food security corresponded to cognitive decline equivalent to an additional 0.7 and 1 year of age per year, respectively. Furthermore, the data from 3037 participants over seven years revealed the effects of food insecurity on cognitive function in Medicare beneficiaries aged 65 and older, indicating that food insecurity was linked to a more rapid decline in executive function, while immediate and delayed memory performance remained unaffected.^18^ On the contrary, in a longitudinal study, Lu et al found that individuals facing food insecurity experienced a faster rate of memory decline, leading to an additional 0.67 years of memory aging over a decade compared to those who were food-secure.^22^

 Limited access to adequate food is a crucial factor that significantly impacts health and nutrition outcomes, particularly among the elderly population.^30,31^ Several mechanisms have been proposed to link food insecurity to AD and related dementias in older adults. Food insecurity, stemming from financial constraints, limits access to nutritious foods, leading to poor dietary quality, skipped meals, and reduced meal sizes, ultimately exacerbating malnutrition.^18,20^ Malnutrition itself is a critical factor, as deficiencies in essential nutrients, such as vitamins and minerals, are known to negatively affect brain function and cognitive health.^20,32,33^ In addition to dietary impacts, food insecurity can trigger chronic stress and anxiety, activating stress pathways that are harmful to brain health over time.^18,22,33^ The constant strain from food insecurity can elevate levels of cortisol and other stress hormones, which have been linked to neurodegeneration and memory decline.^20,34^

 The primary strengths of our study are a case-control design, a high participation rate among subjects, and an analysis adjusted for confounding variables. In addition, to ensure that dietary changes resulting from AD or its diagnosis did not influence the findings, this study included only patients in the early stages of the disease who had received their diagnosis within the past six months. However, our research has several limitations. Firstly, the case-control design introduces the possibility of recall bias, as individuals diagnosed with AD and their caregivers may recall past dietary habits differently, potentially leading to an overestimation of the observed associations. Secondly, retrospective case-control studies are prone to selection bias. To address this concern in our study, we mitigated the risk by achieving high participation rates and recruiting control participants from a range of health centers across the city, ensuring representation from diverse socio-economic groups. A notable limitation of our study was the limited precision of the results, which is largely due to the relatively small sample size. Future prospective studies are necessary to further clarify the role of different levels of food insecurity in predicting AD risk and to establish a plan to minimize detrimental effects. Eventually, AD is a progressive condition that may commence several years before the initial symptoms manifest, whereas food insecurity has been measured after an AD diagnosis. This discrepancy may represent a limitation in our study, as it does not account for the potential temporal mismatch between the chronic nature of AD and the state of food insecurity. However, overall studies conducted to date suggest that diet may be recalled with acceptable levels of misclassifications up to approximately 10 years.^35,36^ Furthermore, to address this limitation, we only included patients whose caregivers had knowledge of the patient’s pre-diagnosis diet. Specifically, we asked the caregiver to respond based on the patient’s condition prior to diagnosis.

**Highlights**

- Individuals experiencing food insecurity had a 2.80 times higher chance of developing Alzheimer’s than those who were food secure.
- Among female subjects, food insecurity increased the odds of AD by 3.45 times.
- For individuals under 65, food insecurity increased the likelihood of AD nearly three-fold.

## Conclusion

 In summary, our findings indicated that food insecurity might be associated with an increased risk of AD. Recognizing and understanding the impact of food insecurity on health can help alleviate its burden. However, further research is required to explore the relationship between food insecurity and other types of neurological disorders or health conditions and provide a clearer and more comprehensive picture of its effects.

## Acknowledgments

 The authors gratefully thank the Iran Dementia and Alzheimer’s Association, the Deputy of Health from Shahid Beheshti and Iran Universities of Medical Sciences, and all the participants and staff for their assistance and cooperation.

## Competing Interests

 The authors declared no personal or financial conflict of interests.

## Ethical Approval

 The researchers guaranteed the confidentiality of respondents’ identity and data. The participants were assured that the information would be used for research purposes. A written informed consent form was obtained from all participants before enrolment in the study. The Ethics Board of the National Nutrition and Food Technology Research Institute of Shahid Beheshti University of Medical Sciences approved the study protocol (approval No. IR.SBMU.NNFTRI.REC.1402.027.

## Funding

 This study was funded by the National Nutrition and Food Technology Research Institute, Shahid Beheshti University of Medical Sciences, Tehran, Iran.
